# Supplementary material for: High and Sustained Ex Vivo Frequency but Altered Phenotype of SARS-CoV-2-Specific CD4+ T-Cells in an Anti-CD20-Treated Patient with Prolonged COVID-19
Source: Viruses. 2022 Jun 10;14(6):1265. doi: 10.3390/v14061265 (PMC9228841; doi:10.3390/v14061265)
Supplement: Supplementary file 1 [file viruses-14-01265-s001.zip › viruses-1730063-supplementary.pdf]

Table S1. Flow cytometry antibody panel used for ex vivo DRB1*11 Tetramer staining.

Fluorochrome	Antigen	Clone	Supplier	Dilution
BUV737	CD4	SK3	BD	1:300
BV711	CD39	A1	BioLegend	1:100
BV650	CCR7	G043H7	BioLegend	1:100
BV605	CD73	AD2	BioLegend	1:100
BV510	CD45RA	HI100	BioLegend	1:300
BV421	LAG-3	11C3C65	BioLegend	1:100
PerCP-Cy5.5	CXCR5	RF8B2	BD	1:60
PE-Cy7	TIGIT	A15153G	BioLegend	1:60
PE-Dazzle594	PD-1	EH12.2H7	BioLegend	1:300
AlexaFluor700	CD3	UCHT1	BioLegend	1:75
AlexaFluor647	CD127	A019D5	BioLegend	1:300
APC-Cy7	CD14	63D3	BioLegend	1:600
APC-Cy7	CD19	HIB19	BioLegend	1:600

Table S2. Peptide pool of 10 non-Spike peptides used for ex vivo ELISpot assay. The peptide highlighted in bold font (M protein aa145-160) covers major parts of the aa sequence loaded on the DRB1*11 Tetramer.

protein and amino acids	aa sequence
N protein aa50-56	SWFTALTQHGKEDLK
N protein aa80-95	DDQIGYYRRATRRIR
N protein aa85-100	YYRRATRRIRGGDGK
N protein aa125-140	NKDGIIWVATEGALN
N protein aa215-230	DAALALLLLDRLNQL
N protein aa345-360	FKDQVILLNKHIDAY
N protein aa350-365	ILLNKHIDAYKTFPP
**M protein aa145-160**	**RGHLRIAGHHLGRCD**
M protein aa175-190	LSYYKLGASQRVAGD
E protein aa55-70	FYVYSRVKNLNSSRV

Table S3. Flow cytometry antibody panel used for intracellular cytokine staining.

Fluorochrome	Antigen	Clone	Supplier	Dilution
BUV737	IL-2	MQ1-17H12	BD	1:60
BUV395	TNF-α	Mab11	BD	1:75
BV711	CD8	RPA-T8	BioLegend	1:150
BV650	CD45RA	HI100	BioLegend	1:600
BV510	CD4	SK3	BioLegend	1:300
BV421	CCR7	G043H7	BioLegend	1:300
PE-Cy7	IFN-γ	4S.B3	BD	1:60
AlexaFluor700	CD3	UCHT1	BioLegend	1:75
APC-Cy7	CD14	63D3	BioLegend	1:600
APC-Cy7	CD19	HIB19	BioLegend	1:600

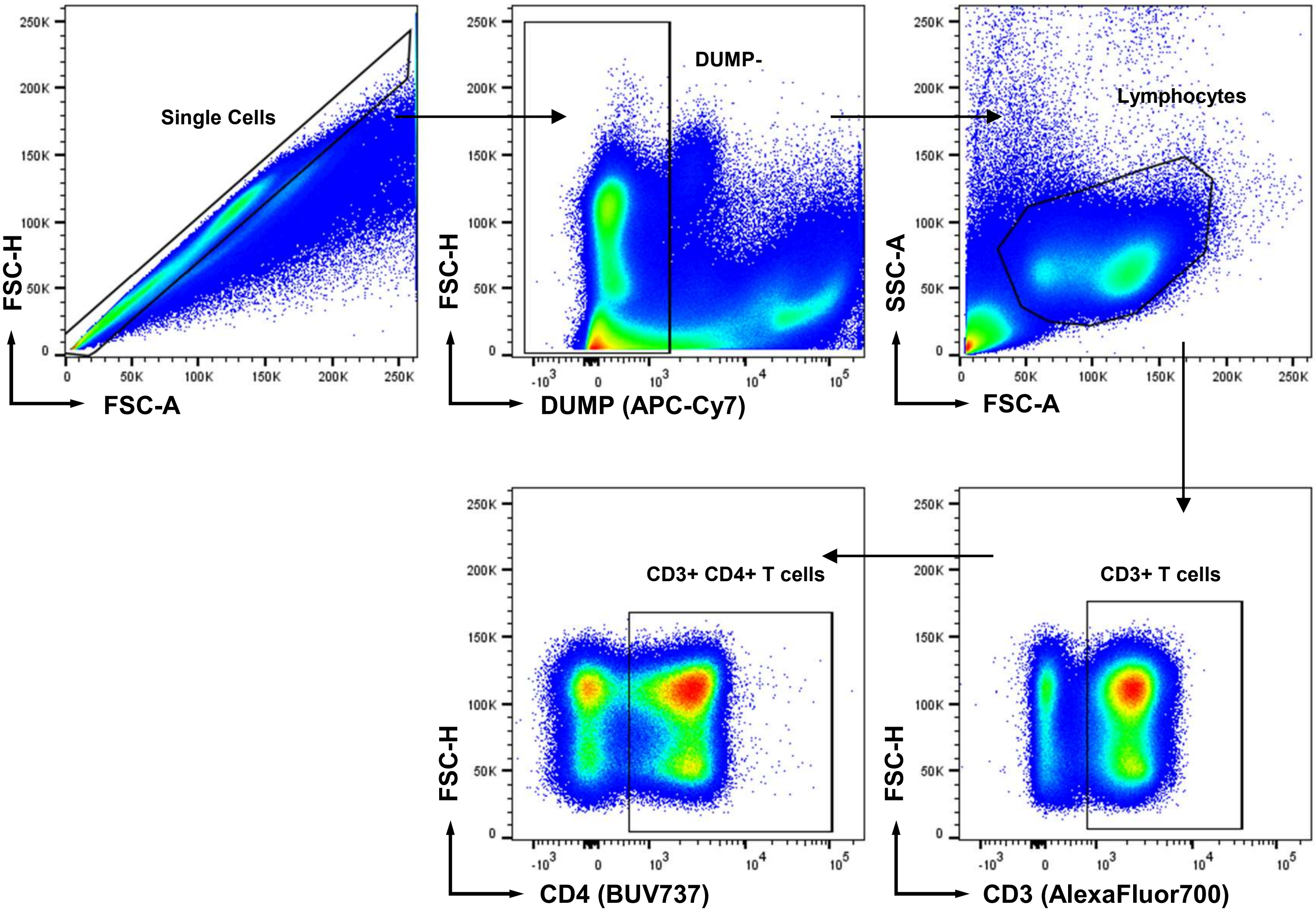

Figure S1. Gating strategy for the identification of CD4+ T cells. First, doublets as well as Monocytes (CD14+), B cells (CD19+) and dead cells (Zombie NIR+) were excluded. In the remaining DUMP- events, CD4+ T cells were identified as CD3 and CD4 positive lymphocytes.

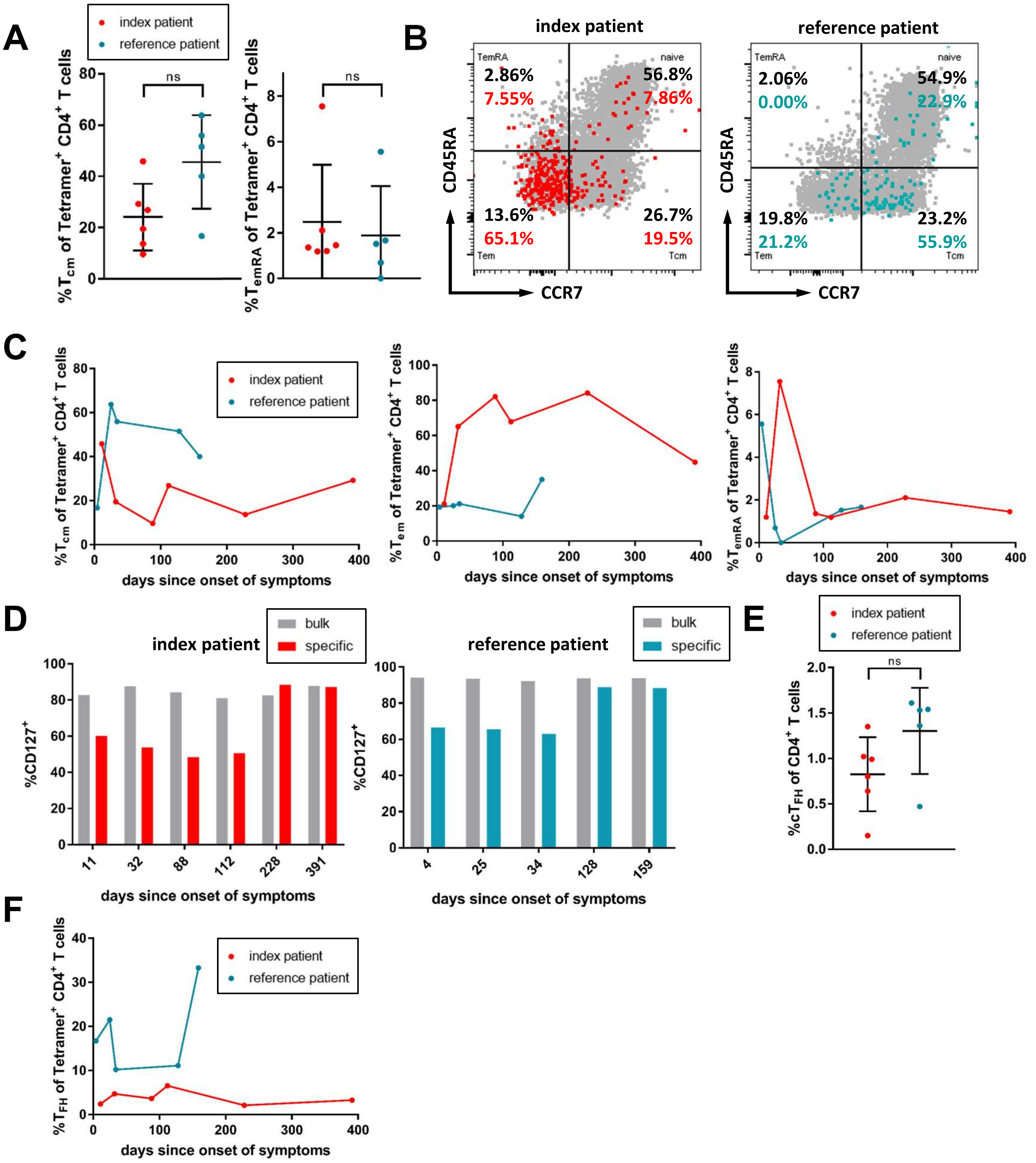

Figure S2. Differentiation status among SARS-CoV-2 specific CD4+ T cells. In the comparison of the memory subsets within the SARS-CoV-2 specific CD4+ T cells, the index patient compared to the reference patient shows slightly reduced frequencies of the Tcm phenotype and slightly elevated frequencies of the TemRA phenotype (A). Representative flow cytometry plots from day 32 (index patient; left) and day 34 (reference patient; right) of the memory phenotype support this observation (B). Longitudinal assessment of the memory phenotype of the SARS-CoV-2-specific CD4+ T cells during the investigated period (C). CD127 expression on bulk (grey) and SARS-CoV-2 specific CD4+ T cells of the index (red) and reference patient (blue) (D). Frequencies of bulk T cells with cTfh phenotype do not differ between the index and reference patient (E). Longitudinal assessment of SARS-CoV-2 specific CD4+ T cells with cTfh phenotype(F).

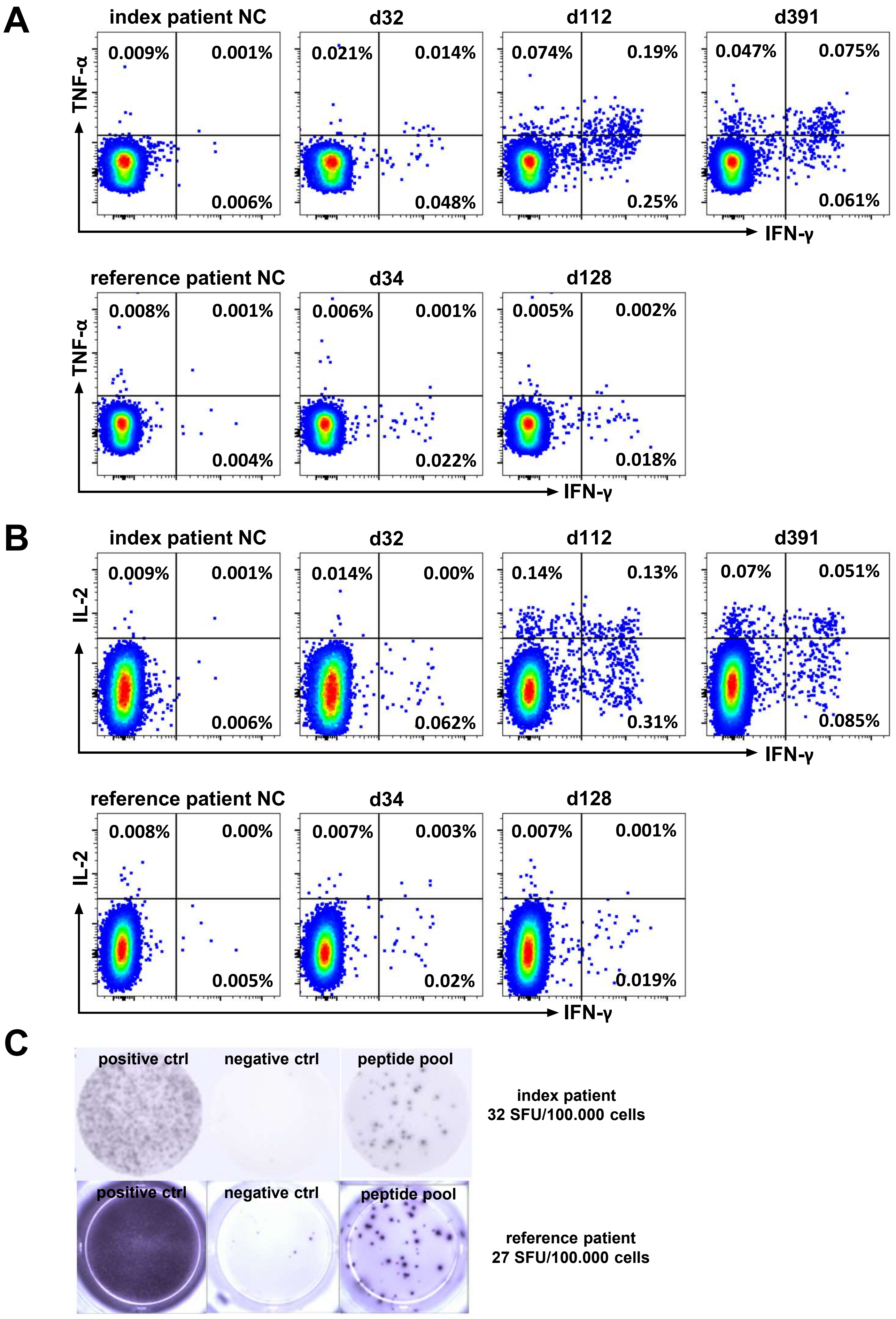

Figure S3. Cytokine responses to a pool of non-spike peptides. Dot plots from intracellular cytokine staining reveal higher frequencies of CD4+ T cells co-producing IFN-γ and TNF-α (A) or IFN-γ and IL-2 (B) after peptide pool stimulation in the index patient compared to the reference patient. NC indicates negative controls. IFN-γ ELISpot shows relevant IFN-γ response 17 months (index patient, upper row) and 8 months (reference patient, lower row) after the onset of symptoms (C).

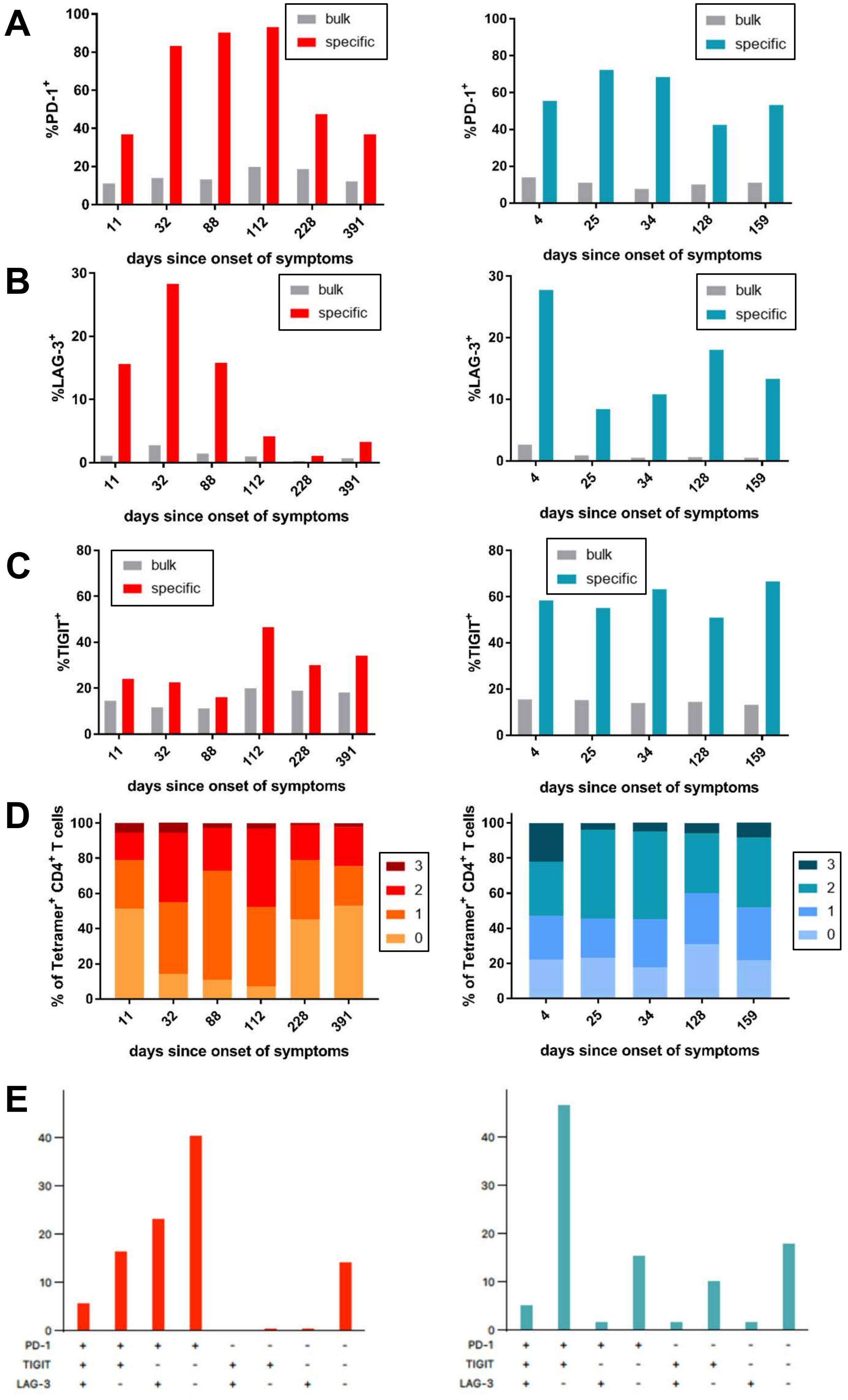

Figure S4. Co-inhibitory receptor expression and co-expression on bulk and SARS-CoV-2 specific CD4+ T cells. Expression of PD-1 (A), LAG-3 (B) and TIGIT (C) on bulk (grey) and SARS-CoV-2 specific CD4+ T cells during the investigated period for the index patient (left; red) and the reference patient (right; blue). Analysis of the number of co-expressed co-inhibitory receptors (PD-1, LAG-3, TIGIT) on SARS-CoV-2 specific CD4+ T cells of the index patient (left; red) and the reference patient (right; blue) at different time points (D). Examplary co-Expression analysis of PD-1, LAG-3 and TIGIT on SARS-CoV-2 specific CD4+ T cellsof the index patient (day 32; red) and the reference patient (day 34; blue).

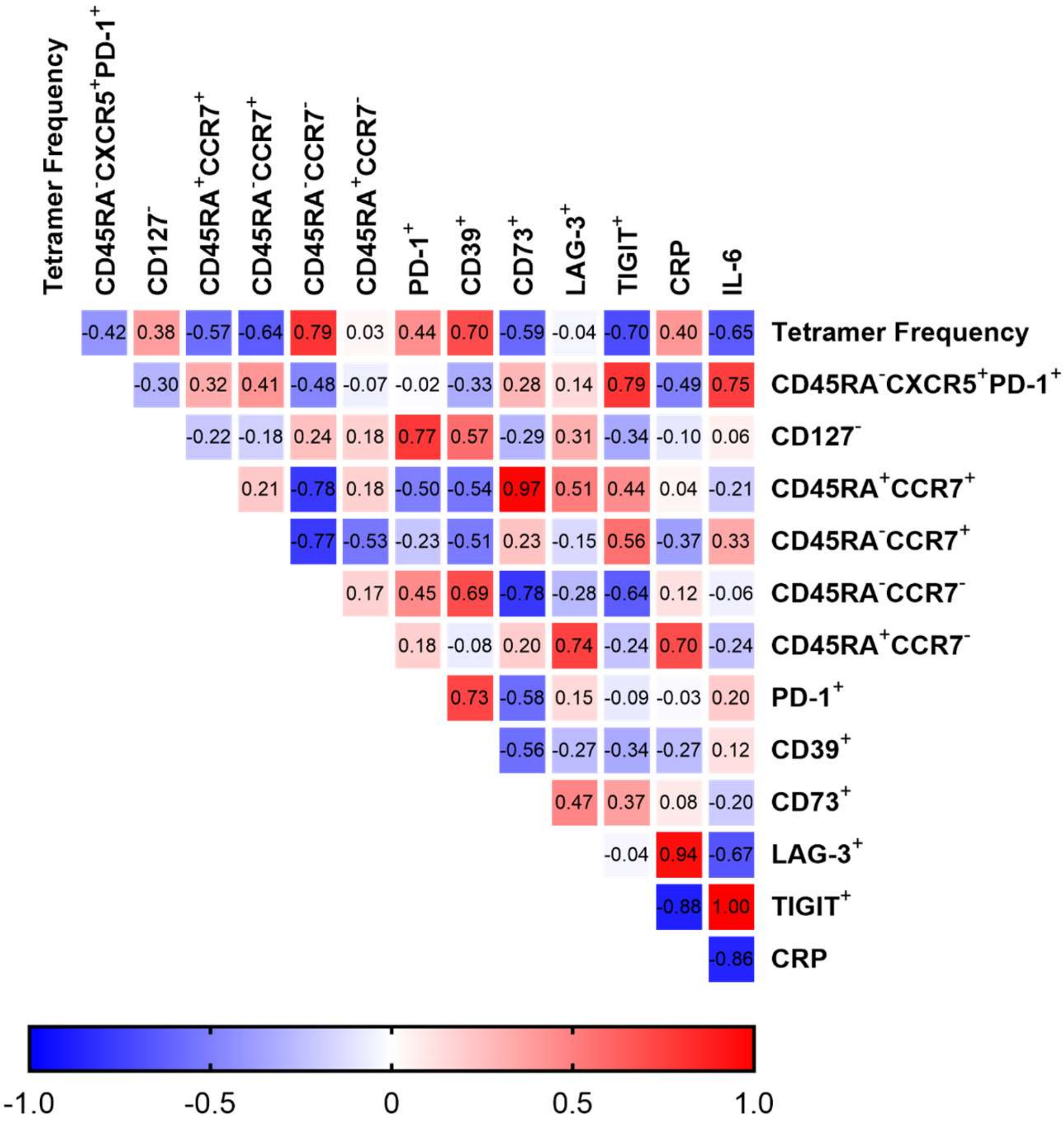

Figure S5. Correlogram of the Tetramer+ CD4+ T cell phenotype. The phenotype of SARS-CoV-2-specific CD4+ T cells at individual time points was correlated. Pearson r was rounded to two decimals.
